# Choroidal and Retinal OCTA Parameters after Scleral Buckling

**DOI:** 10.18502/jovr.v19i4.14432

**Published:** 2024-12-31

**Authors:** Siamak Moradian, Mahmoud Dehghan, Morteza Borandeh Seifi, Mohsen Dastmardi, Fatemeh Suri

**Affiliations:** ^1^Ophthalmic Research Center, Research Institute for Ophthalmology and Vision Science, Shahid Beheshti University of Medical Sciences, Tehran, Iran; ^3^Siamak Moradian: https://orcid.org/0000-0002-5328-7565

**Keywords:** Choroidal Vascularity Index, Optical Coherence Tomography Angiography, Rhegmatogenous Retinal Detachment, Scleral Buckling

## Abstract

**Purpose:**

To evaluate choroidal structure and vasculature in eyes following scleral buckling (SB) for rhegmatogenous retinal detachment (RRD) compared with fellow eyes and control eyes.

**Methods:**

This retrospective observational study was performed on 84 eyes: 32 eyes in the study group consisting of patients who had undergone SB due to RRD, 32 fellow eyes of the same patients, and 20 normal control eyes. Choroidal structures and optical coherence tomography angiography (OCTA) parameters were measured and compared among the three study groups.

**Results:**

In the study group, the mean total choroidal area (0.6816 ± 0.03188 mm^2^) was comparable to the fellow eyes (0.7199 ± 0.03056 mm^2^) and the control group (0.7154 ± 0.17160 mm^2^). Mean outer luminal area was significantly lower in the study group (0.4089 ± 0.00442 mm^2^) than in the fellow eyes (0.4437 ± 0.00701 mm^2^) and the control group (0.4475 ± 0.00442 mm^2^) (*P *= 0.042, and 0.047, respectively). The choroidal vascularity index (CVI) and OCTA parameters were not significantly different in eyes with prior SB compared to the fellow eyes and the control group.

**Conclusion:**

SB does not significantly affect CVI, choroidal vasculature, and OCTA parameters.

##  INTRODUCTION

Rhegmatogenous retinal detachment (RRD) refers to the separation of the neurosensory retina from the retinal pigment epithelium (RPE) due to retinal break which causes vision loss.^[1]^ The two main treatments for this condition are scleral buckling (SB) and pars plana vitrectomy. Pars plana vitrectomy consists of surgically removing the vitreous and reattaching the retina with different techniques.^[2]^ SB is considered the gold standard treatment of RRD and has been performed for about 60 years. In this procedure, episcleral implants are placed on the surface of the sclera to reduce the vitreous traction on the retina and close retinal breaks. SB involves the application of multiple surgical techniques and the selection of various types of buckle elements. Examples include radial versus circumferential and segmental versus encircling buckles, which are selected based on several factors such as the number and location of retinal tears.^[3]^ Some of the common complications associated with SB include refractive changes, intrusion or extrusion and infection of buckle elements, anterior and posterior segment ischemia, and choroidal detachment.^[4]^

SB can also change choroidal blood flow and the hemodynamic structure of the eye.^[5]^ This surgical procedure may cause choroidal congestion and reduce the blood flow of the choroid in the short term while forming veno-venous anastomosis in the choroid in the long term.^[6,7]^ Additionally, SB can affect other choroidal parameters by increasing choroidal thickness and the total choroidal area (TCA), which is the sum of the luminal area (LA) and stromal area (SA).^[8]^ Changes in the choroidal parameters can affect visual function. Thus, it has been shown that the value of the choroidal vascularity index (CVI), particularly within the inner choroidal layers, is positively related to the visual acuity (VA) in patients with retinitis pigmentosa. It was also revealed that the higher the CVI value, the better the VA values, and the larger the LA, the better the visual function.^[9]^ Nevertheless, while several studies have investigated changes in choroidal parameters after SB, they have reported conflicting results.^[6,7][10][11][12]^

In recent years, enhanced depth imaging optical coherence tomography (EDI-OCT) has improved the accuracy of studying choroidal structures, including the measurement of CVI and other choroidal parameters.^[10]^ In this study, we aimed to evaluate choroidal structures and retinal optical coherence tomography angiography (OCTA) parameters in eyes with prior SB surgery as compared to fellow eyes of the same patients and a control group.

##  METHODS

A retrospective observational study was performed on 52 patients (84 eyes) in three groups. The study group included 32 eyes of 32 patients who underwent SB surgery. Of these, 5 eyes had macula-on and 27 eyes had macula-off status. Additionally, an encircling band was placed for 10 eyes, and a segmental buckle was inserted for 22 eyes [Tables 1 & 2].

The fellow eye group included the contralateral eye of each patient in the study group. Given the potential for inherent choroidal changes in both eyes of patients with RRD and because this was a retrospective study without any preoperative OCTA parameters, 20 normal healthy eyes were also included in the study as the control group. All participants provided written informed consent. The study protocol was approved by the Research Ethics Committee of the School of Medicine, Shahid Beheshti University of Medical Sciences, Tehran, Iran, and it adhered to the tenets of the Declaration of Helsinki (ethics code: IR.SBMU.MSP.REC.1401.232).

The participants in the control group had a best-corrected visual acuity (BCVA) ≥ 20/30, intraocular pressure of < 21 mmHg, and no history of ocular surgery, intraocular injections, or retinal diseases. The inclusion criteria for the study group were age < 18 years and successful retinal reattachment after one SB surgery. On the other hand, the exclusion criteria were as follows: history of any ocular surgeries except for SB, diabetic retinopathy, uveitis, optic nerve atrophy, glaucoma, presence of any pathological disorders in the anterior chamber or macula, refractive error greater than ± 6 diopters, and systemic medical conditions such as diabetes, hypertension, or vascular diseases.

Historical findings were recorded and a complete eye examination was conducted. BCVA was measured using the Snellen chart, and the results were converted to logMAR for statistical evaluation. The average follow-up time for study group was 32.03 months [Tables 1 & 2].

OCTA and spectral domain EDI–OCT were performed (Heidelberg Engineering, Heidelberg, Germany) on all included eyes. To minimize the effect of the diurnal choroidal changes on the results, we performed EDI–OCT between 11 AM and 1 PM for all the examinations. Besides, the same operator performed all scans.

Moreover, the center of the fovea (1.5 mm) was scanned horizontally for EDI–OCT images, and then the images were analyzed using ImageJ software according to a previously described protocol.^[13]^

Subfoveal choroidal thickness (SFCT), central macular thickness (CMT), and other retinal layer thickness values were all measured manually with the caliper tool embedded in the Spectralis software.

**Figure 1:**
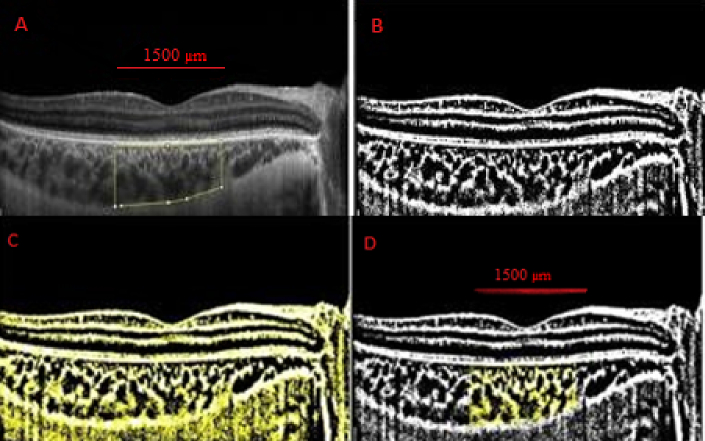
Images of a representative eye that underwent scleral buckling. Choroidal vascular index was calculated through binarization of EDI–OCT images. (A) Total choroidal area is demonstrated by tracing the borders of the inner choroid (yellow line). (B) The image is binarized with Niblack's auto local threshold. (C) Dark and light pixels were separated using the color threshold tool. (D) Dark pixels from the outer border of the RPE–Bruch's membrane layer to the choroid–scleral border were hand-picked using the color threshold tool, and they represent the luminal area (yellow dots). In addition, the bright pixel area from the outer border of the RPE–Bruch's membrane layer to the choroid–sclera border represents the stromal area (the choroidal vascular index was obtained by dividing the luminal area by the total choroidal area).

The TCA, LA, and SA parameters were measured as follows. The TCA in the EDI–OCT image, 1500 μ m wide horizontally and centered on the fovea, was determined by measuring the distance from the outer boundary of the RPE–Bruch's membrane layer to the choroid–sclera junction. The choroidal areas were segmented using the Threshold Tool. The light pixel area was considered ‎as SA and the dark pixel area as LA. The LA and SA were ‎calculated by summing the distance of the two pixels. The CVI was calculated as a proportion of LA to TCA [Figure 1]. The inner and outer choroidal areas were defined as described by Hirashima et al.^[14]^ The inner choroidal area, centered on the fovea, was determined by ImageJ software with a thickness of 70 μ m and a width of 1500 μ m [Figure 1].Furthermore, vascular density of superficial and deep capillary plexus, foveal avascular zone area, and flow void area of choriocapillaris were all measured by OCTA (Optovue, Inc., Fremont, CA, USA). Choroidal and OCTA parameters were compared among the three groups.

### Statistical Analysis

To evaluate the correlation between age and changes in OCT and OCTA parameters, we used the Pearson correlation coefficient and categorized patients into six age groups (17–26, 27–35, 36–44, 45–53, 54–62, and 63–72 years).

Data were analyzed using SPSS version 19. The normal distribution of continuous variables was checked using the Kolmogorov–Smirnov test. One-way analysis of variance (ANOVA) was used to compare variables with a normal distribution between groups. Pearson correlation coefficient and chi-squared tests were performed to evaluate the relationship between examination parameters with age categories and gender, respectively. For all analyses, P < 0.05 was considered statistically significant.

##  RESULTS

We evaluated 84 eyes of 52 subjects with a mean age of 46.61 ± 13.05 years. Of the 84 eyes, 32 were in the study group consisting of patients who had undergone SB due to RRD and 32 eyes were the fellow eyes of the same patients. Most of the patients (n = 20, 62.5%) were male. Besides, there were 20 normal eyes as controls. There was a significant difference in preoperative BCVA between macula-on and macula-off groups (1.71 ± 0.79 and 0.42 ± 0.23 logMAR, respectively; P < 0.001), whereas postoperative BCVA was not significantly different between these two groups (P = 0.83). The mean BCVA of patients before surgery and after surgery is shown in Table 1. The preoperative mean axial length in the study group (25.03 ± 1.17 mm) was significantly higher than in the fellow eyes (24.7 ± 1.15 mm, P = 0.009). The mean follow-up period for patients in the study group was 32.03 ± 5.1 months. SFCT was not significantly different between macula-off and macula-on groups (P = 0.68).

### Demographic Characteristics

**Table 1:** Participants' demographic characteristics.

		**Groups**							
		** Macula-off **			**Macula-on**				
		**Study (scleral buckle)**	**Fellow eye ${\;}^{Ω}$ **	**Control ${\;}^{Ω}$ **	**Study (scleral buckle)**	**Fellow eye**	**Control**	***P*** **-value β **	**Pairwise ${\;}^{@}$ ** ***P*** **-value**
Gender	Female	7 (25.9%)	__	__	1 (20.0%)	8 (25.0%)	6 (30.0%)		
	Male	20 (74.1%)	__	__	4 (80.0%)	24 (75.0%)	14 (70.0%)		
Laterality	OD	15 (55.6%)	__	__	1 (20.0%)	16 (50.0%)	10 (50.0%)		
	OS	12 (44.4%)	__	__	4 (80.0%)	16 (50.0%)	10 (50.0%)		
Scleral buckling material	Segmental sponge505	19 (70.4%)	__	__	3 (60.0%)	0 (0.0%)	0 (0.0%)		
	Band +Tire276	8 (29.6%)	__	__	2 (40.0%)	0 (0.0%)	0 (0.0%)		
Age (yr)		47.48 ± 13.05	__	__	51.4 ± 12.01	48.09 ± 12.79	41.85 ± 13.85	0.171	
Preoperative BCVA ${\;}^{+}$ log MAR		1.71 ± 0.79	__	__	0.42 ± 0.23	0.11 ± 0.21	0.01 ± 0.06	< 0.001	(P1, P2 = 0.001) (P1, P3 < 0.001) (P2, P3 = 0.17)
Postoperative BCVA Log MAR		0.4 ± 0.39	__	__	0.1 ± 0.14	0.12 ± 0.22	__		0.830
Preop.IOP ${\;}^{*}$ (mmHg)		12.19 ± 3.05	__	__	11.8 ± 1.48	13.13 ± 1.84	13 ± 1.65		0.298
Post.OP.IOP ${\;}^{@}$ (mmHg)		13.41 ± 2.94	__	__	12.6 ± 0.55	13.44 ± 2.3	__		0.257
Number of breaks detached retina		1.3 ± 0.61	__	__	1.2 ± 0.45	__	__		
Extent retinal detachment		2.19 ± 0.56	__	__	2.2 ± 0.45	__	__		
Extent scleral Buckling clock		5.69 ± 0.97	__	__	5.3 ± 1.57	__	__		
FU ${\;}^{o}$ (month)		32.56 ± 5.51	__	__	29.2 ± 2.95	32.06 ± 5.38	__		0.257
SE ${\;}^{\Delta }$ (diopter)		-2.81 ± 3.32	__	__	-3.65 ± 4.89	-1.7 ± 3.27	__		0.254
Axial length (mm)		25.02 ± 1.17	__	__	25.1 ± 1.24	24.7 ± 1.15	__	0.475	(P1, P2 = 0.009)
SFCT ${\;}^{**}$ (µm)	__	266.78 ± 71.22	__	__	294 ± 22.97	277.94 ± 63.28	295.33 ± 89.52		0.680
${\;}^{Ω}$ Macula was on in the fellow eye group and the control group without performing any procedure, therefore, the data of each of those two groups was given in one column in the macula-on column and the macula-off section column was left blank; β *P*-value column, we compared the significance or not of the difference between macula-on and macula-off; ${\;}^{@}$ pairwise *P*-value: P1 refers to study group (scleral buckle), P2 refers to fellow eye group, and P3 refers to control eye group; ${\;}^{+}$ BCVA, best corrected visual acuity; ${\;}^{*}$ PreOP.IOP, preoperative intra ocular pressure; ${\;}^{@}$ Post.OP.IOP, postoperative intra ocular pressure; ${\;}^{O}$ FU, follow up; ${\;}^{\Delta }$ SE, spherical equivalent; **SFCT, sub foveal choroidal thickness									

**Table 2:** Retinal thickness and OCTA parameters.

	**Groups**							
	**Macula-off**			**Macula-on**				
	**Study (scleral buckle)**	**Fellow eye ${\;}^{Ω}$ **	**Control ${\;}^{Ω}$ **	**Study (scleral buckle)**	**Fellow eye**	**Control**	***P*** **-valueβ **	**Pairwise ** ***P*** **-value ${\;}^{@}$ **
OCTA ${\;}^{\#}$ scan quality	0.66 ± 0.1	__	__	0.64 ± 0.09	0.69 ± 0.09	0.7 ± 0.12	0.533	
Whole retinal thickness (µm)	278.92 ± 13.96	__	__	286 ± 8.49	280.59 ± 10.13	285.44 ± 11.27	0.343	
Foveal thickness (µm)	268.33 ± 25.01	__	__	262.2 ± 25.99	262.29 ± 24.2	256.83 ± 22.97	0.774	
Parafoveal thickness (µm)	321.58 ± 13.04	__	__	329.2 ± 4.6	325.06 ± 10.84	322.39 ± 13.55	0.498	
Perifoveal thickness (µm)	274.17 ± 14.89	__	__	282 ± 11.34	276.59 ± 10.87	282.78 ± 11.88	0.266	
SCPVD ${\;}^{\Delta }$ whole image (%)	44.8 ± 3.27	__	__	41.84 ± 3.98	45.67 ± 2.53	47.54 ± 4.22	0.010	(P1, P2 = 0.0123) (P1, P3 = 0.009) (P2, P3 = 0.383)
SCPVD fovea (%)	22.98 ± 6.5	__	__	16.62 ± 4	19.48 ± 8.13	20.27 ± 6.45	0.595	
SCPVD parafovea (%)	45.42 ± 3.86	__	__	40.6 ± 4.79	46.64 ± 3.7	47.79 ± 6.37	0.032	(P1, P2 = 0.085) (P1, P3 = 0.029) (P2, P3 > 0.999)
SCPVD perifovea (%)	45.28 ± 3.28	__	__	43.02 ± 4.26	46.31 ± 2.76	48.25 ± 4.36	0.026	(P1, P2 = 0.277) (P1, P3 = 0.026) (P2, P3 = 0.398)
DCPVD ${\;}^{µ}$ whole image (%)	43.38 ± 4.2	__	__	40.78 ± 4.08	43.55 ± 3.79	46.41 ± 4.37	0.018	(P1, P2 = 0.574) (P1, P3 = 0.03) (P2, P3 = 0.14)
DCPVD fovea (%)	38.01 ± 6.71	__	__	32.02 ± 4.89	35.21 ± 7.69	37.94 ± 7.68	0.254	
DCPVD parafovea (%)	49.82 ± 3.75	__	__	49.56 ± 2.26	49.84 ± 4.73	51.74 ± 3.83	0.332	
DCPVD perifovea (%)	43.91 ± 4.45	__	__	40.36 ± 4.8	43.85 ± 4.07	47.17 ± 4.93	0.011	(P1, P2 = 0.425) (P1, P = 30.016) (P2, P3 = 0.114)
FAZ ${\;}^{o}$ area (mm^2^)	0.25 ± 0.08	__	__	0.32 ± 0.08	0.27 ± 0.09	0.25 ± 0.09	0.306	
${\;}^{Ω}$ Macula was on in the fellow eye group and the control group without performing any procedure, therefore, the data of each of those two groups were given in one column in the macula-on column and the macula-off section column was left blank; β *P*-value column, we compared the significance or not of the difference between macula-on and macula-off; ${\;}^{@}$ pairwise *P*-value: P1 refers to study group (scleral buckle) and P2 refers to fellow eye group and P3 refers to control eye group; ${\;}^{\#}$ OCTA, optical coherence tomography angiography; ${\;}^{\Delta }$ SCPVD, superficial capillary plexus vessel density); ${\;}^{µ}$ DCPVD, deep capillary plexus vessel density; ${\;}^{o}$ FAZ, foveal avascular zone.								

**Table 3:** Choroidal structures and OCTA parameters.

	**Groups**									
	**Study**			**Fellow eye**			**Control**			**Pairwise ${\;}^{@}$ **
	**Number**	**Mean**	**SD**	**Number**	**Mean**	**SD**	**Number**	**Mean**	**SD**	***P*** **-value**
Total choroidal area (mm^2^)	32	0.6816	0.03188	32	0.7199	0.03056	20	0.7154	‎0.17160‎	(P1, P2 = 0.385) (P1, P3 = 0.502) (P2, P3 = 0.927)
Total luminal area (mm^2^)	32	0.4830	0.00452	32	0.5169	0.00735	20	0.5189	‎0.12348‎	(P1, P2 = 0.046) (P1, P3 = 0.064) (P2, P3 = 0.917)
Total stromal area (mm^2^)	32	0.3031	0.00344	32	0.2486	0.00843	20	0.2815	‎0.05543‎	(P1, P2 = 0.000) (P1, P3 = 0.072) (P2, P3 = 0.07)
Choroidal vascularity index (%)	32	0.7686	0.04387	32	0.7680	0.04164	20	0.7789	‎0.29408‎	
Inner choroidal area (mm^2^)	32	0.0879	0.00052	32	0.0891	0.00045	20	0.0885	0.00087	
Inner luminal area (mm^2^)	32	0.0741	0.00115	32	0.0732	0.00146	20	0.0714	0.00179	
Inner stromal area (mm^2^)	32	0.0138	0.00106	32	0.0158	0.00134	20	0.0171	0.00133	
Outer choroidal area (mm^2^)	32	0.5937	0.03184	32	0.6309	0.03037	20	0.6479	0.04428	
Outer luminal area (mm^2^)	32	0.4089	0.00442	32	0.4437	0.00701	20	0.4475	0.02802	(P1, P2 = 0.042) (P1, P3 = 0.047) (P2, P3 = 0.843)
Outer stromal area (mm^2^)	32	0.2893	0.00352	32	0.2327	0.00808	20	0.2644	0.01296	
Subfoveal choroidal thickness (µm)	32	271.0313	11.75707	32	277.9375	11.18700	18	295.3333	‎89.5196	
Vascular density of superficial capillary plexus (%)	16	36.4688	1.71332	17	35.4176	1.83552	18	37.9389	‎7.67587‎	
Vascular density of deep capillary plexus (%)	32	24.4148	0.02187	28	24.4095	0.02269	18	24.4697	‎0.09392‎	
Central macular thickness (µm)	29	275.0345	16.50410	29	284.1034	10.60014	1	209.0000		
Outer retina layer thickness (µm)	32	190.7817	2.45940	32	192.9284	2.73954	20	194.6462	3.10850	
Foveal avascular zone (mm^2^)	17	0.2708	0.02104	17	0.2746	0.02290	18	0.2472	0.02185	
Flow void area of choriocapillaris (mm^2^)	32	0.3719	0.03868	32	0.4937	0.04360	20	0.3800	0.05045	(P1, P2 = 0.038) (P1, P3 = 0.903) (P2, P3 = 0.089)
${\;}^{@}$ pairwise *P*-value: P1 refers to study group (scleral buckle) and P2 refers to fellow eye group and P3 refers to control eye group.										

The findings of retinal thickness and OCTA parameters are shown in Table 2.

The chi-square test revealed that the total LA, inner LA, and inner SA were significantly different between the two genders (P < 0.05). Pearson correlation coefficient showed no correlation between the age of the patients and choroidal and OCTA parameters (P > 0.05). The characteristics values of choroidal and OCTA parameters are shown in Table 3.

#### Total choroidal area (TCA)

The mean TCA in the study group (0.6816 ± 0.03188 mm^2^) and control group (0.7154 ± 0.17160 mm^2^) was lower than in the fellow eye group (0.7199 ± 0.03056 mm^2^); however, the results of ANOVA revealed that these differences were not statistically significant (*P* > 0.05) [Table 3].

#### Total luminal area (TLA)

The value of total luminal area (TLA) in the study group (0.4830 ± 0.00452 mm^2^) was significantly lower than in the fellow eye group (0.5169 ± 0.00735 mm^2^). *P*-value?. However, there was no significant difference between the fellow eye group and the control group in this regard [Table 3].

#### Total stromal area (TSA)

The value of the total stromal area (TSA) was significantly higher in the study group (0.3031 ± 0.00344 mm^2^, *P* < 0.001) and control group (0.2815 mm^2^, *P *= 0.007) than in the fellow eye group (0.2486 ± 0.00843 mm^2^) [Table 3].

#### Outer luminal area 

According to the presented data, the value of the outer LA was significantly lower in the study group (0.4089 ± 0.00442 mm^2^) than in the fellow eye group (0.4437 ± 0.00701 mm^2^) and the control group (0.4475 ± 0.00843 mm^2^) (*P *= 0.042 and 0.047, respectively) [Table 3], however, no significant difference was observed between the fellow eye and control groups. This may indicate that SB can reduce the outer LA in operated eyes.

#### Flow void area of the choriocapillaris

According to the results, the mean flow void area of the choriocapillaris in the study group (0.3719 ± 0.03868 mm^2^) was lower than in the fellow eye (0.4937 ± 0.04360 mm^2^) and the control groups (0.38 ± 0.05045 mm^2^), however, the difference was statistically significant only with the fellow eye group (*P* = ?) [Table 3]. The mean vascular density of superficial capillary plexus (SCP) in the SB group was higher than the other two groups, but this difference was not statistically significant (*P* > 0.05).

Based on the results, the values of CVI, SFCT, inner choroidal area, inner LA, inner SA, outer choroidal area, CMT, foveal avascular zone (FAZ) area, and vascular density of deep capillary plexus (DCP) were not significantly different among the three groups (*P* > 0.05).

##  DISCUSSION

In this retrospective observational study, we measured vascular and structural parameters of the choroid after SB surgery based on OCTA and EDI–OCT. In recent years, the study of choroidal structures, including CVI using EDI–OCT has allowed accurate measurement of choroidal parameters.^[10]^

Conflicting results have been reported in previous studies on choroidal vasculature after SB.^[6,7][10][11][12][15][16]^ In a retrospective, cross-sectional study by Iwase et al,^[6]^ the choroidal blood flow and choroidal thickness were measured using laser speckle flowgraphy and OCT after segmental SB in patients with RRD. It was shown that 8 weeks after SB the blood flow was reduced at both the buckled and unbuckled sides but not at the macular area, and then it returned to normal after 12 weeks. They also revealed that the SFCT and LA increased in size one week after SB but it decreased to the normal range in subsequent weeks.^[6]^ However, the results of our study showed that choroidal circulation did not change after SB. Nevertheless, we observed some insignificant changes in OCTA parameters, which might be explained by the fact that we considered all methods of SB surgery and did not focus on just one specific method. The main finding of our study was that the choroidal vascular structures after surgery for SB did not show significant differences compared to those of the same patients' fellow eyes or control eyes.

Our findings revealed that TLA and TSA were significantly lower in the study group as compared to the fellow eyes, although the CVI was comparable. Since CVI is calculated as the proportion of LA to TCA, we observed that TLA and TSA decreased with the same proportion. Conversely, Bernabei et al reported that LA and SA increased after performing encircling SB for patients with RRD. The mean interval between SB and examination in their study was 25.5 ± 16.8 months. Similar to our findings, they revealed that CVI did not change after SB when compared to fellow eyes.^[8]^

The SFCT value did not change significantly among the three groups in the present study. This finding is in line with results reported by Montezuma et al, which demonstrated that choroidal thickness did not change after SB.^[18]^ On the other hand, Odrobina et al revealed that SFCT significantly increased after encircling SB compared to the fellow eyes, and they concluded that the reduction of choroidal blood drainage caused subfoveal choroidal thickening.^[11]^

Several studies revealed that changes in choroidal structures vary depending on the duration of time after SB. For instance, it has been reported that choroidal blood flow decreased after SB in the short term after surgery but returned to baseline in the long term.^[6,9,20]^ This finding supports our observation that there were no significant changes in the choroidal vascular parameters after the mean follow-up period of 32 months. Some studies, however, have indicated that choroidal changes after SB did not return to normal range in the long term.^[21]^

The findings of this study should be considered in the context of some limitations. The nature of the study was retrospective; we did not have the OCT and OCTA parameters of the operated eyes before surgery; therefore, we attempted to overcome this limitation by comparing these data with the information from fellow eyes. We evaluated the patients at only one specific interval after SB, whereas evaluating the choroidal parameters at multiple intervals after surgery could result in more specific findings. A third limitation was the low number of cases we analyzed, which could reduce the impact of the study. The present study did not investigate the effects of multiple SB surgical methods separately on the choroidal parameters; hence, further studies are required to substantiate the findings of the present study.

In summary, CVI and OCTA retinal vascular parameters did not change significantly after SB surgery as compared to the fellow eye and control groups.

##  Financial Support and Sponsorship

None.

##  Conflicts of Interest

None.
